# Effective drug treatment identified by in vivo screening in a transplantable patient-derived xenograft model of chronic myelomonocytic leukemia

**DOI:** 10.1038/s41375-020-0929-3

**Published:** 2020-06-24

**Authors:** Arnold Kloos, Konstantinos Mintzas, Lina Winckler, Razif Gabdoulline, Yasmine Alwie, Nidhi Jyotsana, Nadine Kattre, Renate Schottmann, Michaela Scherr, Charu Gupta, Felix F. Adams, Adrian Schwarzer, Dirk Heckl, Axel Schambach, Suzan Imren, R. Keith Humphries, Arnold Ganser, Felicitas Thol, Michael Heuser

**Affiliations:** 1 Department of Hematology, Hemostasis, Oncology and Stem Cell Transplantation, Hannover Medical School, Hannover, Germany; 2 Department of Biomedical Engineering, Vanderbilt University, Nashville, TN 37235 USA; 3 Institute of Experimental Hematology, Hannover Medical School, Hannover, Germany; 4 Pediatric Hematology and Oncology, Hannover Medical School, Hannover, Germany; 5 Department of Pediatric Hematology and Oncology, Martin-Luther-University Halle-Wittenberg, Halle, Germany; 6 Division of Hematology/Oncology, Boston Children’s Hospital, Harvard Medical School, Boston, MA USA; 7 Department of Pediatrics, University of Washington, Seattle, WA USA; 8 Terry Fox Laboratory, British Columbia Cancer Agency, Vancouver, BC Canada; 9 Department of Medicine, University of British Columbia, Vancouver, BC Canada

**Keywords:** Oncogenesis, Translational research, Preclinical research

## Abstract

To establish novel and effective treatment combinations for chronic myelomonocytic leukemia (CMML) preclinically, we hypothesized that supplementation of CMML cells with the human oncogene *Meningioma 1 (MN1)* promotes expansion and serial transplantability in mice, while maintaining the functional dependencies of these cells on their original genetic profile. Using lentiviral expression of MN1 for oncogenic supplementation and transplanting transduced primary mononuclear CMML cells into immunocompromised mice, we established three serially transplantable CMML-PDX models with disease-related gene mutations that recapitulate the disease in vivo. Ectopic MN1 expression was confirmed to enhance the proliferation of CMML cells, which otherwise did not engraft upon secondary transplantation. Furthermore, MN1-supplemented CMML cells were serially transplantable into recipient mice up to 5 generations. This robust engraftment enabled an in vivo RNA interference screening targeting CMML-related mutated genes including *NRAS*, confirming that their functional relevance is preserved in the presence of MN1. The novel combination treatment with azacitidine and the MEK-inhibitor trametinib additively inhibited ERK-phosphorylation and thus depleted the signal from mutated NRAS. The combination treatment significantly prolonged survival of CMML mice compared to single-agent treatment. Thus, we identified the combination of azacitidine and trametinib as an effective treatment in NRAS-mutated CMML and propose its clinical development.

## Introduction

Chronic myelomonocytic leukemia (CMML) is classified as a hematologic malignancy with myeloproliferative and myelodysplastic features [1] that is diagnosed in approximately 1000 patients per year in the United States [2, 3]. The prognosis varies by risk group and reaches from a median survival of 8 years in the favorable risk group to 16 months in the unfavorable risk group [4]. Similarly, the risk of transformation to acute myeloid leukemia (AML) varies from 0 to 48% among the favorable to high-risk groups [5] and is expected in 16–19% of patients on average [4, 6]. Common cytogenetic aberrations are monosomy 7 and trisomy 8, and somatic mutations in genes that encode epigenetic modifiers (e.g., *TET2*, *ASXL1*, *EZH2*), splicing factors (e.g., *SRSF2*, *SF3B1*, *ZRSF2*), transcription factors (e.g., *RUNX1*), and signaling genes (e.g., *NRAS*, *CBL*, *KRAS*, *JAK2*) are commonly found in CMML patients [5, 7–11].

Treatment options are limited in CMML and include observation, cytoreduction with hydroxyurea, treatment with hypomethylating agents azacitidine or decitabine and allogeneic hematopoietic cell transplantation (alloHCT) in eligible patients. Only alloHCT has a curative intent and resulted in a 3-year overall survival of 31% in single-center experience [12]. Other treatment approaches aim at symptom relief in older patients. A matched-pair analysis of CMML patients treated with azacitidine (*n* = 22) compared to patients treated with hydroxyurea (*n* = 22) resulted in similar median overall survival (7.5 vs 6.2 months, respectively (*p* = 0.251) and thus the benefit of hypomethylating agents over hydroxyurea is unclear [13].

None of the mutations found in CMML patients is unique for the disease. However, *ASXL1*, *TET2* and *SRSF2* mutations occur more frequently in CMML patients (40–50% each) than in other myeloproliferative or myelodysplastic diseases [14]. Interestingly, mutations in signaling pathway genes like *JAK2*, *NRAS* and *CBL* are common in CMML and rare in MDS. CMML cells typically display hypersensitivity to granulocyte-macrophage colony-stimulating factor (GM-CSF) in vitro, which likely contributes to the monocytic phenotype of the disease and is mediated by mutations in signaling genes [15, 16].

In order to better understand the pathophysiology of CMML and to evaluate novel treatment combinations, patient-derived xenograft (PDX) models were developed by transplanting primary patient cells in immunocompromised mice. NOD.Cg-*Prkdc*^*scid*^ *Il2rg*^*tm1Wjl*^/SzJ (NSG) mice transgenic for IL3, SCF and GM-CSF (NSGS) were shown to enhance engraftment of CMML cells likely through expression of GM-CSF [17]. Xenotransplantation of bone marrow or peripheral blood mononuclear cells from 18 CMML patients into NSGS mice gave rise to robust human hematopoietic cell engraftment in almost all cases. However, transplantation into secondary recipients succeeded only in 2 of 5 cases and further serial transplantation was not reported [18]. Similarly, cells from 15 of 16 CMML patients engrafted in primary NSG or NSGS recipient mice, but in secondary NSGS recipients only 3 of 10 samples engrafted with low levels of 2–9% human cells in bone marrow [17].

Overexpression of the transcriptional co-factor MN1 has been observed in a broad spectrum of AML patients [19–23] and has been identified as an independent prognostic marker for AML with normal karyotype. High expression of MN1 is associated with poor prognosis, shorter overall and relapse-free survival, and poor response to treatment [21]. In murine cells human MN1 overexpression induces aggressive, fully penetrant AML through the promotion of leukemic cell self-renewal [24–26], impairment of myeloid differentiation [24, 25], and resistance to all trans retinoic acid-induced differentiation [25]. MN1-induced leukemias are also associated with upregulation of *HOXA* genes and *MEIS1* [27]. Moreover, functional assays have revealed the critical dependence of the MEIS1/AbdB-like HOX protein complex for MN1-induced transformation [27].

We aimed to develop a xenograft model that allows expansion of CMML cells in vivo, serial transplantation for at least 3 generations of mice and functional manipulation of primary CMML cells for an in vivo shRNA screen. We hypothesized that supplementation of CMML cells with a human oncogene will promote expansion and serial transplantability while the functional dependencies of these cells on their genetic profile are maintained. We chose the MN1 oncogene for this approach as transduction of MN1 in CD34 + cord blood cells promotes self-renewal in vitro and engraftment in vivo, but does not induce AML in vivo nor does it enable serial transplantation [28].

To establish a serially transplantable PDX model for CMML we used lentiviral expression of MN1 in primary CMML cells for oncogenic supplementation and evaluated the growth potential of these cells in vivo by serial transplantations, a functional in vivo shRNA screen and a novel combination treatment in vivo.

## Materials and methods

### Patient samples and xenotransplantation

Diagnostic bone marrow or peripheral blood samples were collected after obtaining written informed consent from CMML patients diagnosed and treated at Hannover Medical School [29, 30]. Briefly, mononuclear cells were isolated from patient bone marrow aspirates or peripheral blood by density centrifugation using Biocoll Separating Solution (Biochrom, Berlin, Germany). The cell number was determined and depletion of CD3 positive cells was done following the manufacturer’s protocol (CD3 MicroBeads kit, Miltenyi Biotec, Bergisch Gladbach, Germany). Patient cells were analyzed according to the principles of the Declaration of Helsinki, and the study was approved by the institutional review board of Hannover Medical School. Patient characteristics are listed in Supplementary Table S1. Detailed methods for cell culture, viral vectors and infection of cells, clonogenic progenitor assays, immunoblotting and amplicon-based next-generation sequencing [31, 32] of treated CMML cells are described in the Supplementary Methods.

For xenotransplantation studies, 6- to 8-week-old female NOD.Cg-*Prkdc*^*scid*^ *Il2rg*^*tm1Wjl*^/SzJmice transgenic for human interleukin-3, granulocyte-macrophage-colony-stimulating factor and stem cell factor (NSGS) were bred by the central animal laboratory of Hannover Medical School under pathogen-free conditions. All animal experiments were performed in accordance with the German animal protection law and approved by the Lower Saxony state office for consumer protection, Oldenburg, Germany. Xenotransplantation, engraftment monitoring, treatment and morphologic analysis of CMML cells are described in the Supplementary Methods. Technical details on serial transplantations are provided in Supplementary Table S2.

### Quantitative RT-PCR

Total RNA was extracted from 1 million cells and reverse transcribed as previously described [25]. Quantitative reverse-transcriptase polymerase chain reaction (RT-PCR) was done as previously described using SYBR green (Invitrogen, ThermoFischer Scientific, Schwerte, Germany) for quantification of double-stranded DNA on a StepOne Plus cycler (Applied Biosystems, Darmstadt, Germany). Relative expression was determined with the 2^−∆∆CT^ method using *ABL1* as the housekeeping gene, and expression values were normalized relative to control cells [33]. Used custom primers were shown in Supplementary Table S3.

### Statistical analysis

Data are shown as mean ± standard error of mean (SEM). Student *t* test and Logrank test were used to assess differences between groups and survival curves, respectively. All statistical analysis were performed with GraphPad Prism Version 7 software. *P* < 0.05 was considered statistically significant. The size of the animal cohorts was based on our previous study [34, 35]. All in vitro experiments were performed at least 3 times unless otherwise stated and all attempts of replication were successful.

## Results

### Supplementation of primary CMML cells with MN1 promotes engraftment in vivo

**Fig. 1:**
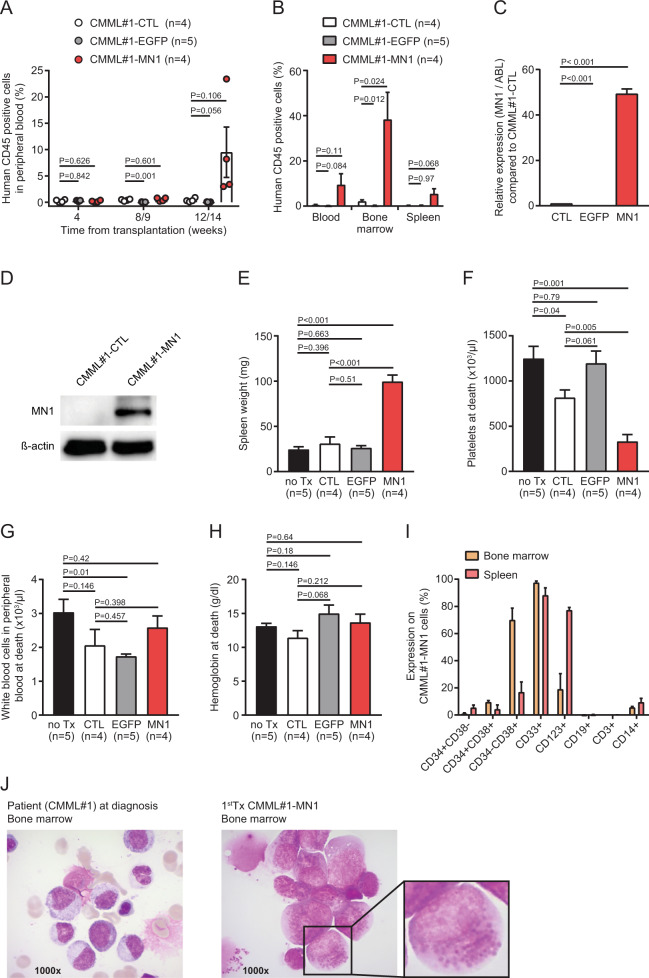
Supplementation of primary CMML cells with MN1 promotes engraftment in vivo. **a** Engraftment of human hematopoietic cells (CD45 positive) in the peripheral blood of primary recipient NSGS mice monitored at the indicated time points. Recipient mice were transplanted with untransduced CMML#1 cells (CMML#1-CTL), EGFP- (control vector) transduced CMML#1 cells (CMML#1-EGFP) or MN1-transduced CMML#1 cells (CMML#1-MN1), (number of analyzed mice is indicated in the figure; mean ± SEM). **b** Engraftment of human CD45 positive cells in peripheral blood, bone marrow and spleen of primary recipient NSGS mice at sacrifice (number of analyzed mice is indicated in the figure; mean ± SEM). **c** Expression levels of MN1 in untransduced CMML#1 patient cells (CMML#1-CTL), EGFP-transduced CMML cells (CMML#1-EGFP) and MN1-transduced CMML cells (CMML#1-MN1). Gene expression was determined by quantitative RT-PCR relative to the housekeeping gene *ABL1* and normalized to gene expression in untransduced CMML#1 patient cells (*n* = 3), (mean ± SEM). **d** Western blot showing MN1 expression in primary bone marrow mononuclear cells from a patient with CMML (CMML#1-CTL) and in their MN1-transduced counterparts isolated from bone marrow of engrafted recipient mice. ß-actin was used in the same blot as loading control. **e** Spleen weight of primary recipient NSGS mice at sacrifice (number of analyzed mice is indicated in the figure; mean ± SEM). **f** Platelet count in peripheral blood of primary recipient NSGS mice at sacrifice (number of analyzed mice is indicated in the figure; mean ± SEM). **g** White blood cell count in peripheral blood of primary recipient NSGS mice at sacrifice (number of analyzed mice is indicated in the figure; mean ± SEM). **h** Hemoglobin level in peripheral blood of primary recipient NSGS mice at sacrifice (number of analyzed mice is indicated in the figure; mean ± SEM). **i** Immunophenotype of engrafted CMML#1 cells from bone marrow and spleen of primary recipient NSGS mice at sacrifice (gated on human CD45 + EGFP + CMML#1-MN1 cells; *n* = 3; mean ± SEM). **j** Morphology from bone marrow smears of the CMML#1 patient at diagnosis and from a transplanted mouse at sacrifice. The enlarged cell shows granules in the cytoplasm that can be identified as lipid granules in cells from later transplantations (see Fig. 3b).

We evaluated the ability of oncogenic supplementation of patient CMML cells with the oncogene MN1 to enhance robust engraftment of CMML in vivo and enable serial transplantation [27, 28]. Transduction of mononuclear cells from bone marrow aspirate of a patient diagnosed with CMML-1 with a control vector (CMML#1-EGFP) or MN1 (CMML#1-MN1) resulted in 77% and 16% efficacy in vitro, respectively (Supplementary Fig. S1A). Intravenous injection of untransduced (CMML#1-CTL) and EGFP-transduced (CMML#1-EGFP) primary CMML#1 cells into sublethally irradiated NSGS recipient mice resulted in minimal or undetectable engraftment of human CD45+ cells for CMML#1-CTL and CMML#1-EGFP cells in peripheral blood 12 weeks after transplantation (Fig. 1a), and in bone marrow 22 weeks after transplantation (0,8% to 3% for CMML#1-CTL, 0,6% for CMML#1-EGFP) and spleen (0.5% for CMML#1-CTL, 0,6% for CMML#1-EGFP) (Fig. 1b). In contrast, MN1- transduced CMML#1 cells initiated robust human hematopoietic engraftment ranging from 3% to 23% in peripheral blood, 21% to 73% in bone marrow and 2% to 12% in spleen 13 weeks after transplantation (Fig. 1a, b). In CMML#1-MN1 cells all human cells expressed EGFP (Supplementary Fig. S1B), and MN1 overexpression was confirmed by RNA and protein expression in engrafted PDX cells compared to primary untransduced CMML#1 cells of the corresponding patient (Fig. 1c, d), suggesting that MN1 was required for engraftment. CMML#1-MN1 mice had enlarged spleens (Fig. 1e) and reduced platelet counts (Fig. 1f), while white blood cell (WBC) counts and hemoglobin levels were similar (Fig. 1g, h) when compared with CMML#1-CTL, CMML#1-EGFP or non-engrafted control (no Tx) groups.

The immunophenotype of engrafted human CD45 and EGFP positive CMML#1-MN1 cells showed a myelo-monocytic differentiation expressing CD33, CD38 and CD14, including progenitor markers CD38 and in few cells CD34 (Fig. 1i). Morphologic analysis of bone marrow from CMML#1-MN1 mice showed myeloid progenitors and monocytic cells similarly to bone marrow smears from the CMML#1 patient at diagnosis and revealed MN1 expression by EGFP fluorescence (Fig. 1j and Supplementary Fig. S1C). In summary, oncogenic supplementation of CMML cells with MN1 enabled engraftment of these cells that otherwise did not engraft in vivo.

### CMML-MN1 cells engraft secondary recipient mice and are serially transplantable

**Fig. 2:**
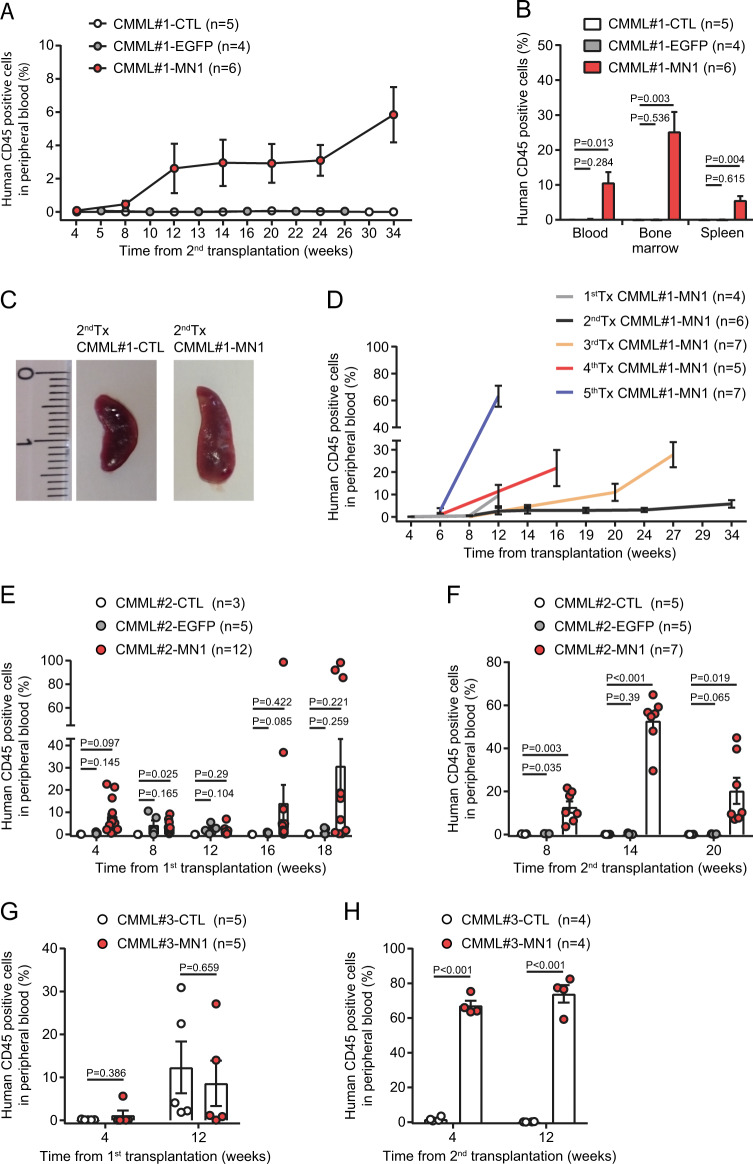
CMML-MN1 cells engraft secondary recipient mice and are serially transplantable. **a** Engraftment of human hematopoietic cells (CD45 positive) in the peripheral blood of secondary recipient NSGS mice monitored at the indicated time points (number of analyzed mice is indicated in the figure; mean ± SEM). **b** Engraftment of CMML#1-MN1 cells in peripheral blood, bone marrow and spleen of secondary recipient NSGS mice at sacrifice (number of analyzed mice is indicated in the figure; mean ± SEM). **c** Representative photographs of spleens from secondary recipient mice transplanted with bone marrow from primary recipient mice which had received either untransduced CMML#1 cells (CMML#1-CTL) or MN1-transduced CMML#1 cells (CMML#1-MN1) (scale bar indicates millimeters). **d** Engraftment of CMML#1-MN1 cells in serial transplantations shown as percentage of human CD45 positive cells detected in peripheral blood at the indicated time points (number of analyzed mice is indicated in the figure; mean ± SEM). **e** Engraftment of CMML#2 cells in the peripheral blood of primary recipient NSGS mice transplanted with untransduced CMML#2 (CMML#2-CTL), EGFP (control vector) transduced CMML#2 (CMML#2-EGFP) or MN1-transduced CMML#2 cells (CMML#2-MN1), (number of analyzed mice is indicated in the figure; mean ± SEM). **f** Engraftment of CMML#2 cells (from Fig. 2e) in the peripheral blood of secondary recipient NSGS mice at the indicated time points (number of analyzed mice is indicated in the figure; mean ± SEM). **g** Engraftment of CMML#3 cells in the peripheral blood of primary recipient NSGS mice transplanted with untransduced CMML#3 (CMML#3-CTL) or MN1-transduced CMML#3 cells (CMML#3-MN1), (number of analyzed mice is indicated in the figure; mean ± SEM). **h** Engraftment of CMML#3 cells (from Fig. 2g) in the peripheral blood of secondary recipient NSGS mice at the indicated time points (number of analyzed mice is indicated in the figure; mean ± SEM).

To evaluate whether CMML#1-MN1 cells can be serially transplanted, CMML#1-CTL, CMML#1-EGFP and CMML#1-MN1 cells from bone marrow of primary recipients were isolated and intravenously transplanted into sublethally irradiated secondary NSGS recipient mice. Only CMML#1-MN1 cells engrafted in secondary recipients (Fig. 2a), and showed mean engraftment of 10%, 25% and 5% of human CD45+ cells in peripheral blood, bone marrow and spleen, respectively, at the time of sacrifice (Fig. 2b). Engrafted CMML#1-MN1 cells had a myeloid phenotype expressing CD33, CD38, and CD123, but lacked expression of lymphoid markers CD3 and CD19 (Supplementary Fig. S1D), and developed splenomegaly (Fig. 2c).

Cryopreserved CMML#1-MN1 cells from bone marrow and spleen of secondary transplanted mice were used as source to initiate serial transplantations in NSGS recipients up to 5 times (Fig. 2d). Serial transplantation resulted in higher engraftment levels in peripheral blood and shorter latency, suggesting that the cells underwent clonal selection.

Cells from two additional CMML patients were used to confirm MN1-enhanced serial engraftment. Detailed patient characteristics are provided in Supplementary Table S1. In the CMML#2 model MN1-transduced cells showed higher engraftment levels in several mice at 16 and 18 weeks after transplantation compared to CTL mice (Fig. 2e). Upon secondary transplantation only MN1-transduced cells engrafted (7% to 44% human CD45+ in peripheral blood, 11% to 98% human CD45+ in bone marrow and 18% to 36% human CD45+ in spleen), while CTL and EGFP-transduced cells did not engraft (Fig. 2f and Supplementary Fig. S2A). In the CMML#3 model we observed comparable robust engraftment of both CMML#3-MN1 and CMML#3-CTL cells at week 12 after transplantation in primary recipient NSGS mice (Fig. 2g). Upon secondary transplantation of spleen and bone marrow cells (65% to 85% human CD45+ cells in 1^st^Tx CMML#3-MN1 and 26% to 47% human CD45+ cells in 1^st^TxCMML#3-CTL mice) only MN1-transduced cells engrafted at high levels (59% to 82% human CD45+ cells in peripheral blood, 93% to 96% in bone marrow and 74% to 88% in spleen), while the CTL group did not engraft secondary recipient NSGS mice (Fig. 2h and Supplementary Fig. S3A). CMML#2-MN1 and CMML#3-MN1 secondary recipient mice had enlarged spleens (Supplementary Figs. S2B and S3B), reduced platelet counts and similar white blood cell counts and hemoglobin levels (Supplementary Figs. S2C-E and S3C-E) when compared with CMML#2-CTL and CMML#2-EGFP, or CMML#3-CTL mice, respectively. Morphology of bone marrow cells isolated from CMML#2 and CMML#3 secondary recipient mice showed myeloid progenitors and monocytic cells similar to bone marrow smears from the CMML#2 and CMML#3 patients (Supplementary Figs. S2F and S3F). Thus, oncogenic supplementation enabled serial transplantation of CMML cells and set the stage for functional in vivo studies in primary patient cells.

### Clonal dominance of an NRAS/NOTCH1 mutated subclone during serial transplantation of CMML#1-MN1 cells

**Fig. 3:**
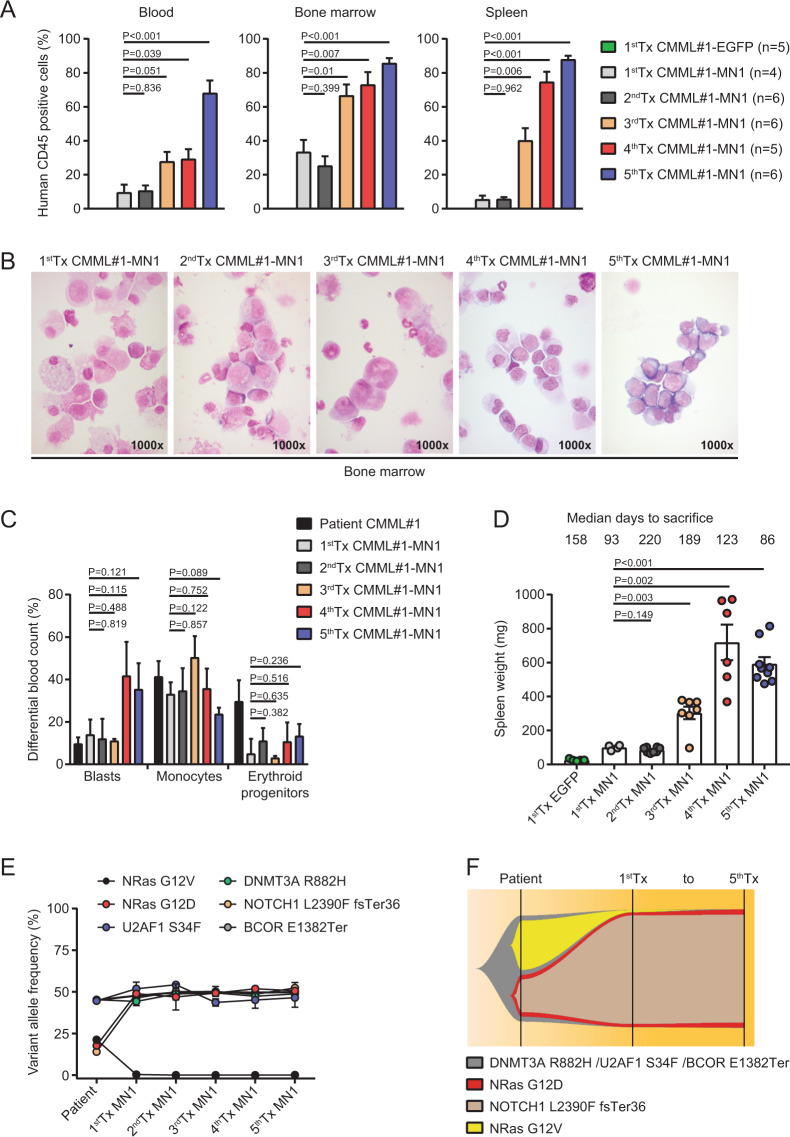
Clonal dominance of an NRAS-mutated subclone during serial transplantation of CMML#1-MN1 cells. **a** Engraftment of human hematopoietic cells in peripheral blood, bone marrow and spleen of NSGS recipient mice from first to fifth serial transplantation (number of analyzed mice is indicated in the figure, mean ± SEM). **b** Morphology from bone marrow smears of engrafted CMML#1-MN1 cells of NSGS recipient mice from first to fifth serial transplantation. **c** Differential blood count quantification of bone marrow smears from the patient (CMML#1) at diagnosis and engrafted CMML#1-MN1 cells in mice from first to fifth transplantation (*n* = 3; mean ± SEM). **d** Spleen weight of primary CMML#1-EGFP recipient mice and CMML#1-MN1 recipient mice from first to fifth serial transplantation at the time of sacrifice (each dot represents one mouse; mean ± SEM). **e** Variant allele frequencies of mutated genes in bone marrow cells of the CMML#1 patient and corresponding CMML#1-MN1 cells engrafted in the bone marrow and spleen of NSGS recipient mice at sacrifice from first to fifth serial transplantation (*n* = 3; mean ± SEM). **f** Graphical representation of clonal evolution of CMML#1 cells from the patient and its corresponding CMML#1-MN1 cells from first to fifth serial transplantation in NSGS recipient mice.

We evaluated the biologic and genetic evolution of CMML#1-MN1 cells during serial transplantation from first to fifth generation. Up to the third transplantation CMML#1-MN1 cells showed preferential engraftment in bone marrow over peripheral blood and spleen (Fig. 3a). In the fourth transplantation engraftment in bone marrow and spleen was comparable and in the fifth transplantation engraftment was equally high in blood, bone marrow and spleen (Fig. 3a). Bone marrow morphology showed a similar monocyte dominated picture compared to the patients’ bone marrow from first to third transplantation, while the blast count increased in the fourth and fifth transplantations above 20%, while the monocytes remained at high levels (Fig. 3b, c). This indicates that upon serial transplantation the most aggressive clones prevail and the disease is more related to AML, while it still reflects the same genetics that were found in the patients’ cells. Spleen weight in sacrificed mice increased over time consistent with engraftment levels in the spleen (Fig. 3d). Genetic profiling of the patient’s cells identified a founding clone characterized by *DNMT3A, U2AF1* and *BCOR* mutations and two subclones with additional mutations. The first subclone was characterized by a *NRAS* G12D mutation and a *NOTCH1* mutation, and the second subclone included a *NRAS* G12V mutation (Fig. 3e). Upon transduction with MN1 and transplantation in vivo only the *NRAS* G12D/*NOTCH1* subclone containing also the *DNMT3A, U2AF1* and *BCOR* mutations engrafted in PDX mice and persisted without further changes up to the fifth transplantation (Fig. 3f and Supplementary Table S4). In summary, serial transplantation led to the selection of a more aggressive leukemic clone that was characterized by mutations recurrently found in CMML and AML patients. Only the *NRAS* G12D/*NOTCH1* mutated subclone stably engrafted in vivo, and its consistent representation in all transplantations suggests that mutations in *DNMT3A*, *U2AF1*, *BCOR*, *NRAS* and *NOTCH1* were required for in vivo proliferation despite the presence of MN1. We found that clonal selection of MN1-transduced CMML cells occurred during engraftment in vivo and not during the ex vivo transduction procedure as shown for the CMML#3-MN1 model (Supplementary Fig. S4).

The known GM-CSF/CSF2 dependency of CMML cells was evaluated for CMML#1-MN1 cells by transplanting cells from the fourth recipient generation into either NSG or NSGS mice. The latter mouse strain produces human GM-CSF besides IL3 and SCF. Upon transplantation of equal cell numbers CMML#1-MN1 cells engrafted much faster in NSGS compared to NSG recipient mice, suggesting that these MN1-transduced cells retained their GM-CSF sensitivity (Supplementary Fig. S5).

### Loss-of-function shRNA-screening identifies functional dependencies in CMML#1-MN1 cells

**Fig. 4:**
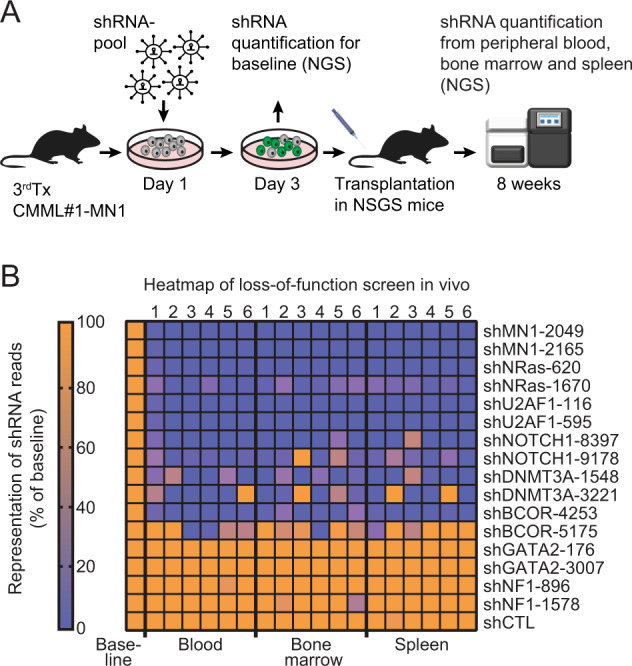
Loss-of-function shRNA-screening identifies functional dependencies in CMML#1-MN1 cells. **a** Experimental outline of loss-of-function shRNA screening showing lentiviral transduction of CMML#1-MN1 cells with a shRNA pool, transplantation into NSGS recipient mice, cell harvest after 8 weeks and quantification of shRNAs by next-generation sequencing. **b** Representation of shRNAs in blood, bone marrow and spleen of NSGS recipient mice at 8 weeks after transplantation compared to baseline shRNA representation (baseline: transduced cells kept in vitro and quantified on day 3), (each column represents one mouse, *n* = 6).

To evaluate the functional importance of the mutated genes and *MN1* we performed a targeted shRNA screening in vivo. Bone marrow cells from the third generation of CMML#1-MN1 mice were harvested and transduced with a shRNA pool consisting of 2 independent shRNAs against 8 genes and 1 control shRNA against luciferase. Six shRNAs targeted the mutated genes in this patient or *MN1*, while 2 shRNAs targeted 2 control genes that are recurrently mutated in myeloid disease (*GATA2* and *NF1*). 48 h after transduction the cells were either transplanted in NSGS mice or harvested for shRNA quantification. Eight weeks after transplantation the cells were harvested from blood, bone marrow and spleen and DNA was extracted from unsorted cells to quantify shRNA copies in total DNA by NGS (Fig. 4a). The shRNAs against the 8 genes were validated in vitro and induced knockdown usually by more than 80% (Supplementary Fig. S6 and Supplementary Table S5). shRNAs against the control genes *luciferase*, *GATA2* and *NF1* were not depleted after transplantation in mice compared to the cells harvested after transduction in vitro (Fig. 4b). Interestingly, the shRNAs against the mutated genes and *MN1* were strongly depleted in most mice and cell sources (blood, bone marrow, spleen) after in vivo transplantation except one of the two shRNAs against *BCOR* (Fig. 4b). This analysis suggests that at least four mutated genes are required for the survival of CMML-MN1 cells in vivo in addition to *MN1* and shows that our model can be used to evaluate the specific contribution and biology of each mutated gene despite the ectopic expression of the *MN1* oncogene.

### The MEK-inhibitor trametinib prolongs survival of CMML-MN1 mice when combined with azacitidine

**Fig. 5:**
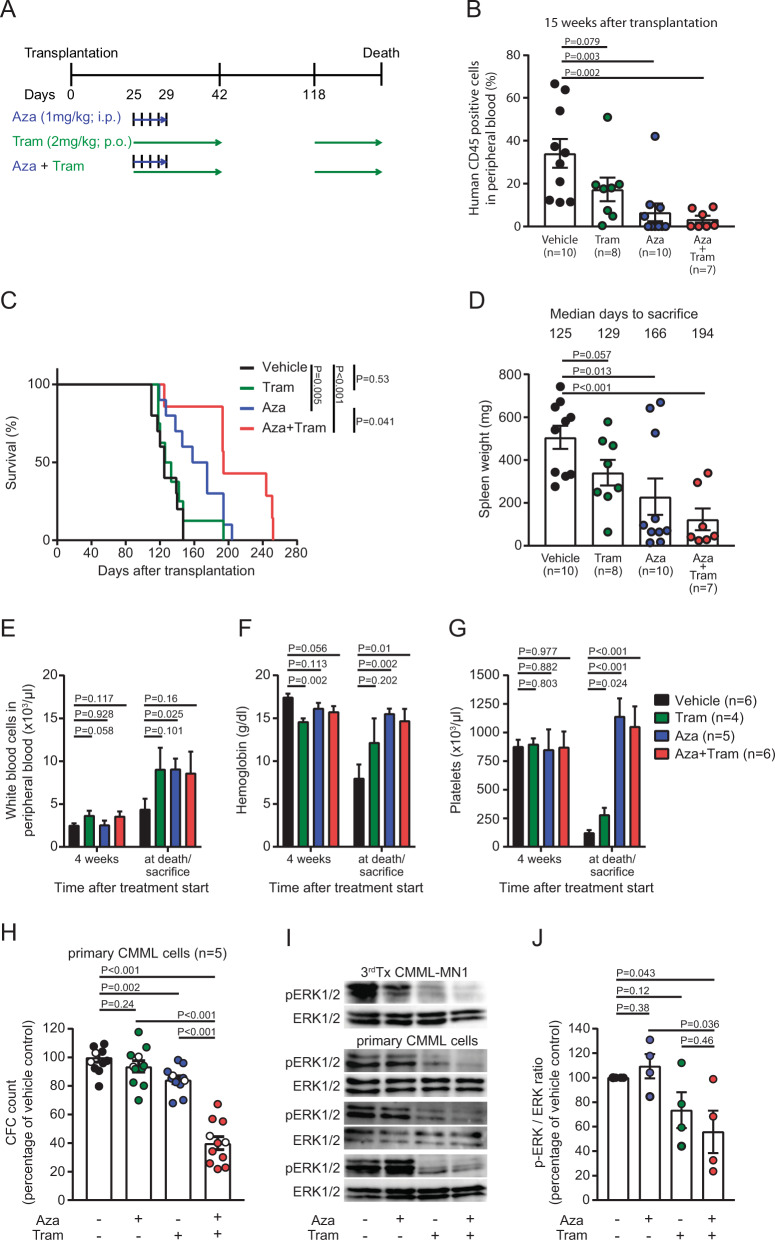
The MEK-inhibitor trametinib prolongs survival of CMML#1-MN1 mice when combined with azacitidine. **a** Schematic representation of the treatment regimens (Aza, azacitidine; Tram, trametinib). **b** Monitoring of CMML burden in recipient mice at week 15 after transplantation (each dot represents one mouse; mean ± SEM). **c** Survival of NSGS recipient mice engrafted with CMML#1-MN1 cells and treated with vehicle (*n* = 10), trametinib (*n* = 8), azacitidine (*n* = 10) and the combination of trametinib+azacitidine (*n* = 7). **d** Spleen weight of NSGS recipient mice engrafted with CMML#1-MN1 cells and treated with vehicle, trametinib, azacitidine and the combination of trametinib+azacitidine at sacrifice (each dot represents one mouse; mean ± SEM). **e** White blood cell count in peripheral blood of CMML#1-MN1 bearing mice treated with vehicle, trametinib, azacitidine and the combination of trametinib+azacitidine 4 weeks after treatment start and at sacrifice (number of individual mice is indicated in the figure; mean ± SEM). **f** Hemoglobin levels in peripheral blood of CMML#1-MN1 bearing mice treated with vehicle, trametinib, azacitidine and the combination of trametinib+azacitidine 4 weeks after treatment start and at sacrifice (number of individual mice is indicated in the figure; mean ± SEM). **g** Platelet count in peripheral blood of CMML#1-MN1 bearing mice treated with vehicle, trametinib, azacitidine and the combination of trametinib+azacitidine 4 weeks after treatment start and at sacrifice (number of individual mice is indicated in the figure; mean ± SEM). **h** Effect of the combination treatment in five different CMML patients (unrelated to the CMML#1-MN1 cells). Primary CMML cells were treated with vehicle, azacitidine (500 nM), trametinib (20 nM) or the combination of azacitidine (500 nM) + trametinib (20 nM) and plated in duplicate in CFC media. After 20 days colonies were counted and expressed as percentage of the vehicle-treated cells. White dots within all groups represent cells from a patient without mutation in a signaling gene (*n* = 5, mean ± SEM). **i** Immunoblotting for p-ERK and ERK in azacitidine/trametinib- and vehicle-treated CMML patients (*n* = 3) and CMML#1-MN1 (*n* = 1) cells from third recipient mice at 6 h after treatment. **j** Average p-ERK-to-ERK ratio from immunoblots shown in Fig. 5i as percentage of vehicle-treated CMML cells (*n* = 4, mean ± SEM).

Hypomethylating agents are used in CMML patients with proliferative disease who are not eligible for alloHCT. As the NRAS-mutated subclone engrafted efficiently in NSGS mice and knockdown of NRAS depleted CMML#1 cells, we hypothesized that the combination of the MEK-inhibitor trametinib, which inhibits the signaling pathway downstream of NRAS, with azacitidine more efficiently inhibits CMML development than either drug alone. We transplanted CMML#1-MN1 cells from the fifth transplantation in NSGS mice and confirmed similar engraftment of human CD45+ EGFP+ cells in peripheral blood 2 weeks after transplantation (Supplementary Fig. S7A). Treatment was started 4 weeks after transplantation with azacitidine (1 mg/kg i.p. for 5 days) and/or trametinib (2 mg/kg p.o. for 18 days). At this time we assume similar engraftment levels between the indicated groups, as 4 weeks after the start of treatment engraftment was still comparable between the treatment groups (Supplementary Fig. S7B). Trametinib treatment was interrupted until day 118 and then continued until the end of the experiment (Fig. 5a). Engraftment monitoring at week 15 after transplantation revealed diminished engraftment in peripheral blood of mice treated with azacitidine (6% CD45+ cells) and the combination of azacitidine+trametinib (3% CD45+ cells) as compared to trametinib treated (17% CD45+ cells) and vehicle-treated (34% CD45+ cells) mice (Fig. 5b and Supplementary Fig. S7B). Trametinib treated mice had similar survival as vehicle-treated mice, while azacitidine treated mice showed improved survival (Fig. 5c). Interestingly, mice treated with the combination of trametinib/azacitidine survived significantly longer than vehicle treated, azacitidine treated or trametinib treated mice alone (median survival for vehicle 125 days, azacitidine 166 days, trametinib 129 days and trametinib/azacitidine 194 days, Fig. 5c, *P* = 0.041). Engraftment of CMML-MN1 cells was significantly lower in azacitidine and trametinib/azacitidine treated groups compared to vehicle control (Supplementary Fig. S7C). The weight of the spleens was lower in the single and combination treatment groups compared to the vehicle group despite a longer latency to sacrifice (Fig. 5d). At the end of the treatment, WBC counts and hemoglobin counts were decreased in the vehicle group but remained in the normal range for azacitidine and trametinib/azacitidine treated groups. Platelets were increased in azacitidine and trametinib/azacitidine treated groups when compared with vehicle or trametinib treated mice (Fig. 5e–g).

To evaluate whether the additive effect of trametinib and azacitidine was also found in primary cells from CMML patients we performed CFC assays from peripheral blood or bone marrow cells of five CMML patients (Supplementary Table S1). The combination treatment reduced the CFC count significantly stronger compared to either azacitidine or trametinib monotherapy (Fig. 5h). In line with this finding, phosphorylated ERK (pERK) was most effectively inhibited in CMML#1-MN1 cells harvested from NSGS mice as well as primary CMML cells by the combination treatment compared to single-agent azacitidine, trametinib or vehicle treatment (Fig. 5i, j). In summary, the combination of the MEK-inhibitor trametinib and a hypomethylating agent prolonged survival in the CMML#1-MN1 model by additively inhibiting pERK signaling.

## Discussion

We developed patient-derived CMML models that recapitulate the disease in vivo and allowed us to demonstrate that 1) ectopic MN1 expression enhances the engraftment and proliferation of cells that otherwise do not engraft in mice, 2) the functional relevance of CMML-related gene mutations is preserved in the presence of MN1, 3) MEK/ERK signaling is important for in vivo proliferation of CMML cells, 4) the serial transplantability of the MN1-CMML cells enables functional RNAi screening in vivo and 5) the combination of azacitidine with trametinib is an effective combination treatment in CMML#1-MN1 cells in vivo and primary CMML cells in vitro.

It was shown in recent years that CMML cells readily engraft in immunocompromised mice by intrafemoral or intravenous injection and are transplantable in secondary recipient mice in some cases [17, 18]. However, transplantation in secondary recipients was inconsistent and no tertiary transplantation was reported. We chose the MN1 oncogene to enhance the engraftment of CMML cells as MN1 enhances self-renewal and blocks differentiation of CD34+ cord blood cells in vitro and induces a myeloproliferative disease, but not AML, in vivo. Only by combining *MN1* with another oncogene, *NUP98-HOXD13*, could cord blood cells be transformed to AML and serially transplanted [28]. Here we confirm our hypothesis that the mutations present in CMML cells collaborate with MN1 and maintain their functional relevance. So far, only expression of the fusion genes *MLL-AF9* and *MLL-ENL* were able to fully transform normal human hematopoietic stem cells [36–39]. The incomplete transformative capacity of MN1 may be ideally suited to enhance the proliferative capacity of malignant cells and enable their investigation in vivo.

The robust engraftment of MN1-CMML cells allowed us to perform in vivo RNA interference targeting the mutated genes in our patient. The cells were clearly dependent on MN1, but also on *NRAS*, *NOTCH1*, *U2AF1* and *DNMT3A*, while shRNAs against the non-mutated genes *GATA2* and *NF1* and the control shRNA were not depleting. The results for *BCOR* were inconsistent between the two targeting shRNAs. Mutations in signaling gens like *NRAS* are found in 30% of CMML patients [7, 9] and the *NRAS*/*NOTCH1* mutated subclone in our patient was selected during the transplantation procedure and persisted throughout all 5 serial transplantations. Ricci et al. [40] underscored the importance of RAS pathway mutations by showing that they induce a CMML-like disease in mice. *NOTCH1* mutations have not been reported in CMML, but mutations in NOTCH pathway genes were found in 5 of 47 CMML patients [41]. Klinakis et al. showed that deletion of at least NOTCH1 and NOTCH2 induced a CMML-like disease in mice [41]. It remains unclear whether the *NOTCH1* mutation in our model was a loss-of-function or rather gain-of-function mutation, as inhibiting *NOTCH1* in our experiments by shRNA depleted the CMML cells in vivo. *U2AF1* mutations occur in approximately 7% of CMML patients [42]. U2AF1 mutated cells had a competitive disadvantage in mouse models compared to U2AF1 wildtype cells. The combination of mutated *U2AF1* with *RUNX1* induced AML in mice, but 2 of 3 established AMLs lost the *U2AF1* mutation [43]. It is thus surprising that the *U2AF1* mutation was maintained in our model over 5 generations, which represents a faithful model to study the impact of *U2AF1* mutations in vivo. Knockdown of *U2AF1* resulted in depletion of CMML cells in vivo in our study. A gain-of-function has been suspected for *U2AF1* mutations, as they occur only at 2 hotspots in this gene, but catastrophic depletion of the splicing factor SRSF2 has also led to cell depletion [44], and thus the nature of our *U2AF1* mutation remains unclear. *DNMT3A* mutations occur in approximately 5% of CMML patients [45]. The Arg882His mutation found in our patient indicates an origin of the disease in clonal hematopoiesis of indeterminate potential [46]. This mutation impairs the methyltransferase activity of DNMT3A [47] and loss of DNMT3A leads to enhanced self-renewal of hematopoietic stem cells and expansion of the DNMT3A null clone [48]. It was therefore unexpected that shRNA-mediated knockdown of *DNMT3A* depleted CMML cells in vivo. However, DNMT3A depletion in hepatocellular carcinoma cells resulted in reduced proliferation [49].

As RAS-pathway mutations are frequent in CMML and the MEK-inhibitor trametinib is approved for melanoma and shows single-agent clinical activity in RAS mutated patients with myeloid malignancies including CMML [50], we tested the combination treatment of trametinib and azacitidine in vivo. The reduced engraftment at 15 weeks, reduced spleen weight at death and the significantly prolonged survival compared to azacitidine monotherapy suggests that this combination is at least additive in NRAS-mutated CMML cells. The additive effect was confirmed in CFC assays with primary, non-transduced patient cells from five CMML patients and by the strong synergistic inhibition of phosphorylated ERK (p-ERK) in MN1-CMML and primary patient cells. All five patients included in the CFC assay lacked mutations in NRAS, but four of the five patients had a mutation in signaling genes that regulate the same pathway (2 patients with *CBL* mutations, one patient with a *cKit* mutation and one patient with a *JAK2* mutation). This suggests that CMML cells may be sensitive to trametinib independent of *NRAS* mutations, as they have frequent comutations in other signaling genes. So far, the combination of azacitidine with trametinib has not been tested in CMML patients with or without RAS-pathway mutations, but our data suggest that this combination should be evaluated in a phase II clinical trial.

In summary, we established a transplantable CMML model with *NRAS*, *NOTCH1*, *DNMT3A*, *U2AF1* and *BCOR* mutations and validated the functional importance of mutated NRAS. We identified the combination of azacitidine and trametinib as an effective treatment in NRAS-mutated CMML and suggest its clinical development to eventually improve the outcome of CMML patients.

## Supplementary information

Supplemental data
