# Supplementary material for: Effective drug treatment identified by in vivo screening in a transplantable patient-derived xenograft model of chronic myelomonocytic leukemia
Source: Leukemia. 2020 Jun 24;34(11):2951–63. doi: 10.1038/s41375-020-0929-3 (PMC7116758; doi:10.1038/s41375-020-0929-3)
Supplement: Supplementary file 1 — Supplemental data [file 41375_2020_929_MOESM1_ESM.docx]

## Effective drug treatment identified by in vivo screening in a transplantable patient-derived xenograft model of chronic myelomonocytic leukemia

## Supplemental Data

## Supplementary Methods

### Cell culture

K562 cells were cultured in RPMI 1640 medium (Gibco, Thermofisher Scientific, Bremen, Germany) supplemented with 10% fetal bovine serum (FBS) and incubated at 37°C with 5% CO_2_ in the humidified atmosphere and were regularly tested for mycoplasma contamination. For drug treatment and lentiviral infection, patient-derived CMML cells and primary CMML cells were cultured and pre-stimulated in Iscove’s modified Dulbecco’s medium (IMDM, StemCell Technologies, Cologne, Germany) supplemented with 20% bovine serum albumin, insulin and transferrin (BIT 9500, StemCell Technologies, Cologne, Germany), 10^-4^ M 2-mercaptoethanol (Sigma-Aldrich, Munich, Germany), 2 mM L-Glutamine (Gibco, Thermofisher Scientific, Bremen, Germany), 20 ng/ml human IL-6, 20 ng/ml human IL-3, 20 ng/ml human granulocyte colony-stimulating factor (G-CSF), 100 ng/ml human stem cell factor (SCF), 50 ng/ml thrombopoietin (TPO) and 100 ng/ml FLT3-ligand (all from PeproTech, Hamburg, Germany) and incubated at 37°C with 5% CO_2_ in the humidified atmosphere.

### Viral vectors and infection of cells

The lentiviral vector pCCL-c-MNDUS-MN1-pgkEGFP (11.8 kilobases) was used for MN1 expression. Lentiviral control vector pCCL-c-MNDUS-pgkEGFP (7.8 kilobases) was generated by removing the MN1 expression sequence of the pCCL-c-MNDUS-MN1-pgkEGFP vector. Both vectors were used for lentivirus production by transient transfection of 293T cells. Lentiviral particles were concentrated by ultracentrifugation.

Primary CMML cells were resuspended in culture medium with 5µg/ml protamine sulfate, centrifuged at 1500 rpm for 30 minutes in a 12 well plate coated with 5µg/cm^2^ fibronectin (RetroNectin, Takara Bio Inc, St Germain en Laye, France) and preloaded with concentrated MN1 and EGFP lentivirus and cultured at 37°C for 24 hours.

shRNAs targeting the human genes *MN1*, *NRAS*, *U2AF1*, *NOTCH1*, *DNMT3A*, *BCOR*, *GATA2* and *NF1* were designed and cloned into the lentiviral plasmid pLKO5d.SFFV.EGFP.miR-N as described by Adams et al.([1](#_ENREF_1)) In short, 67 bp oligonucleotides encoding shRNA sequences for cloning into the miR-N cassette were purchased from Integrated DNA Technologies (IDT, Leuven, Belgium). Oligonucleotides were phosphorylated by T4 PNK (NEB, Frankfurt, Germany) at 37 °C for 45 min, heated to 95 °C for 2.5 minutes and annealed by cooling to 22 °C at 0.1 °C/second. These oligonucleotides were diluted 1:500 and ligated into the BsmBI (NEB, Frankfurt, Germany) restriction site of the linearized lentiviral backbone by standard cloning procedures.

Lentiviral particles encoding single shRNA-expressing constructs directed against the human genes mentioned above were produced by transient transfection of 293T cells. K562 cells were resuspended in fresh lentivirus-containing supernatant supplemented with protamine sulfate (5µg/ml, Sigma-Aldrich, Munich, Germany) every 24 hours for 3 days. Transduced K562 cells were sorted for EGFP prior to RNA extraction.

To generate the lentiviral shRNA library, several shRNA vectors were scaled up and pooled prior transfection of 293T cells. Engrafted CMML#1-MN1 cells were isolated from mice and transduced with the lentiviral shRNA library by spin inoculation as described above. After lentiviral infection, all cells were washed three times with PBS prior in vivo or in vitro experiments.

### Xenotransplantation, treatment and monitoring of mice

One million lentivirally transduced (CMML-MN1 or CMML-EGFP) and untransduced (CMML-CTL) primary mononuclear CMML cells were transplanted intravenously in the lateral tail vein of sublethally (2,5 Gy) irradiated NSGS mice. For therapeutic studies one million patient-derived CMML#1 cells isolated from bone marrow or spleen of CMML#1-MN1 bearing mice were retransplanted intravenously in the tail vein of sublethally irradiated NSGS mice. Treatment was initiated 4 weeks after transplantation with either vehicle, azacitidine (1mg/kg, intraperitoneally), trametinib (2mg/kg, oral gavage) or the combination of azacitidine and trametinib as indicated in the results section.

Complete blood counts were measured using an ABC Vet Automated Blood counter (Scil animal care company GmbH, Viernheim, Germany). Spleen weight and complete blood counts in peripheral blood, bone marrow and spleen were measured at sacrifice. Survival of treated mice was monitored daily. Bone marrow or spleen cells from engrafted mice were retransplanted in NSGS recipient mice up to 5 times.

**Neither randomization, nor blinding was used in animal experiments since all animal experiments were performed with a homogeneous strain, age, and similar variance.** Animals which died before the start of treatment due to engraftment failure were excluded from the study.

### Clonogenic progenitor assay

Colony-forming cell units were assayed in methylcellulose (Methocult H4100; StemCell Technologies, Cologne, Germany) supplemented with 10 ng/mL IL3, 10 ng/mL GM-CSF, 50 ng/mL SCF, 50 ng/mL FLT3-ligand and 3 U/mL EPO (PeproTech, Hamburg, Germany). Vehicle, azacitidine (500 nM), trametinib (20 nM) or the combination of azacitidine (500 nM) with trametinib (20 nM) were added to methylcellulose containing 10 thousand human CMML cells and were plated in duplicate. Colonies were evaluated microscopically 20 days after plating by standard criteria.

### Immunoblotting

For MN1 immunoblotting whole cell lysates from CMML#1-MN1 PDX cells and primary CMML#1 cells were prepared with lysis buffer (20 mM HEPES, pH 7.5, 0.4 M NaCl; 1mM EDTA, 1 mM EGTA, 1 mM DTT) supplemented with one mini complete protease inhibitor cocktail tablet (Roche Diagnostics, Mannheim, Germany) following sonication for 20 seconds at 30% amplitude (Sonopuls, Bandelin electronic, Berlin, Germany).

For immunoblotting of ERK and phosphorylated ERK, CMML-MN1 PDX and primary CMML cells were treated with either vehicle, azacitidine (1 µM), trametinib (20 nM) or the combination of azacitidine (1 µM) and trametinib (20 nM). 6 hours after treatment 3 million cells were collected, washed with PBS and resuspended in lysis buffer supplemented with protease and phosphatase inhibitors (Pierce RIPA buffer, Halt Protease & Phosphatase inhibitor Cocktail, all from Thermo Scientific, Rockford, USA) by gentle shaking on ice for 20 minutes. After clearing lysates at 10,000 rpm for 10 minutes at 4°C, cellular protein concentrations were determined using the Pierce BCA Protein Assay Kit (Thermo Scientific, Rockford, USA).

Protein containing supernatants were adjusted for equal amounts and separated by SDS-PAGE, transferred to a PVDF membrane, blocked and immunoblotted with antibodies against ERK (1:1000, p44/42 MAPK (ERK1/2, 137F5), phosphorylated ERK (1:1000, Phospho-p44/42 MAPK (ERK1/2, Thr202/Tyr204), all from Cell Signaling Technology, Frankfurt, Germany, ß-actin (1:5000, monoclonal anti-ß-actin clone AC-74, Sigma-Aldrich, Munich, Germany and MN1 (1:500, anti-MN1 ab112916, Abcam, Cambridge, UK). The secondary horseradish peroxidase-conjugated antibodies anti-rabbit (NA934) and anti-mouse (NA931V), both from GE Amersham, Freiburg, Germany) were used for chemiluminescent protein detection by Clarity Western ECL Substrate (Biorad, Munich, Germany) with a ChemiDoc MP Imaging System (Bio-Rad, Munich, Germany).

### Next-generation sequencing for clonal tracking and shRNA screening

Mutations in patient cells were evaluated by an amplicon-based next generation sequencing (NGS) approach. DNA sequencing libraries were prepared from samples at diagnosis with a custom TruSight myeloid sequencing panel according to the manufacturers’ instructions (Illumina, San Diego, CA). The panel includes 46 entire genes or hotspots recurrently found in myeloid leukemias (Supplementary Table S6). The details of the myeloid panel sequencing and related data analysis were described previously.([2](#_ENREF_2), [3](#_ENREF_3))

Mutations in CMML-MN1 xenografts from consecutive transplantations were assessed with an error correcting sequencing approach established previously for MRD (measurable residual disease) analysis.([3](#_ENREF_3)) For the MRD approach approximately 100 bp genomic regions around the known mutation positions were amplified using specially designed primers (Supplementary Table S7). Sequencing depth was 1,070-28,700 reads (median 3,800 reads). The VAF was calculated from Sanger sequencing data by dividing the peak height of the variant nucleotide by the height of the consensus nucleotide.

Fishplots displaying changes in clonal structure over time were generated with the R package fishplot ([4](#_ENREF_4)) using the VAFs of the mutations of patient cells or CMML-MN1 cells from consecutive transplantations at the indicated time points.

For the in vivo RNA intereference screen we quantified the copy number of each shRNA in DNA extracted from various cell populations by NGS. Quantification of shRNA integrates followed a modified protocol for measurable residual disease assessment by NGS. In the first PCR lentiviral integrates including the shRNA-miR-N cassette were amplified by primers complementary to EGFP and WPRE sequences of the lentiviral backbone. The PCR reaction was performed with Q5 DNA polymerase (NEB, Frankfurt, Germany) with the following conditions: 98°C for 30 seconds, 30 cycles of 98°C for 10 seconds, 61°C for 30 seconds and 72°C for 20 seconds, and a final step of 72°C for 2 minutes. The PCR products were cleaned up using the QIAquick PCR Purification Kit (Qiagen, Hilden, Germany). 200 nanograms of purified DNA served as templates for miR-N specific primers fused to a 20 basepair unique common sequence to amplify shRNA sequences in the second PCR (98°C for 30 seconds, 30 cycles of 98°C for 10 seconds, 62°C for 30 seconds, and 72°C for 12 seconds, and a final step of 72°C for 2 minuntes). The amplicons were purified with AMPure XP beads (Beckman Coulter, Krefeld, Germany) by mixing beads and PCR product at a ratio of 0.9:1 for 20 minutes. For size selection, the PCR products containing supernatant was again mixed with beads at a ratio of 0.21:1 for 7 minutes and the bead-bound DNA was gently washed with ethanol and solubilized with H_2_O. Size selected DNA was barcoded in the third PCR with common sequence specific primers fused to a unique multiplex identifier (MID), a sequence complementary to the custom sequencing primer and the Illumina adapters P5 or P7. The conditions for the third PCR reaction were 98°C for 30 seconds, 25 cycles of 98°C for 10 seconds, 67°C for 50 seconds and 72°C for 12 seconds, and a final step with 72°C for 2 minutes. AMPure XP beads were added to the PCR product at a ratio of 0.8:1 for 20 minutes and bead-bound DNA was washed with ethanol and solubilized in elution buffer (Qiagen, Hilden, Germany). Eluted DNA underwent an additional purification step using the Gene Read Size Selection Kit according to the manufacturer´s protocol (Qiagen, Hilden, Germany). DNA from up to 40 samples was then pooled at equimolar concentrations. 600 µl of a 20 pM DNA solution was added to a MiSeq reagent v3 kit and 251 cycles were sequenced in both directions on a MiSeq sequencer (Illumina, San Diego, CA) using custom forward and reverse sequencing primers. The median number of quantified shRNA reads in transduced CMML-MN1 cells was 40,500 per sample. All primers used for amplicon-based NGS are described in Supplementary Table S8.

The shRNA screening results were analyzed by counting the individual shRNA sequences obtained from NGS. 73-basepair shRNA sequence signatures were defined from constant and variable loop sequences flanked by 5 basepairs. Pairwise differences between signatures were sufficiently large, i.e. the editing distance was more than 9, which was defined as the number of nucleotide differences between the signatures. One mismatch was allowed upon parsing of the signatures. As control we counted the signatures allowing 0 or 2 mismatches. The error rate was less than 0.2% at each nucleotide position, therefore 1 mismatch in 73 positions should occur in less than 14.6% of sequences. Further, less than 2.2% (<14.6% of 14.6%) of the sequences should have 2 mismatches. Thus, shRNA sequences were accepted if we observed such a behavior for each individual shRNA.

### Engraftment monitoring and morphology

Engraftment of human CMML cells was monitored by regular blood sampling and flow cytometric analysis using a FACS Calibur cytometer (BD Biosciences, Heidelberg, Germany) and a CytoFLEX cytometer (Beckman Coulter, Krefeld, Germany). Following erythrocyte lysis (BD Pharm Lyse, BD Biosciences, Heidelberg, Germany), peripheral blood, bone marrow and spleen cells were stained for engraftment monitoring and immunophenotyping with anti-human-antibodies CD45-PE (H130, CAT 304039), CD33-APC (WM53, CAT 303408) from BioLegend (Koblenz, Germany), CD38-PE (HB-7, CAT 345806), CD34-APC (8G12, CAT 345804), CD14-PE (MφP9, CAT 345785), CD123-APC (7G3, CAT 560087), CD3-PE (UCHT1, CAT 555333) and CD19-APC (HIB19, CAT 555415) from BD Biosciences (Heidelberg, Germany). Flow cytometry data were analyzed using FlowJo software (V10.0.7, TreeStar).

For morphologic analysis CMML-PDX cells were isolated from mouse bone marrow by immunomagnetic selection (Mouse Cell Depletion Kit, Miltenyi Biotec, Bergisch Gladbach, Germany). The proportion of MN1-transduced cells in these enriched cell populations was determined by EGFP-fluorescence on unstained cytospin preparations using an Olympus BX60 (Olympus, Tokyo, Japan) microscope with a 40x/0.75 numerical aperture objective and Cell Imaging software (Olympus Life Science Europa GmbH, Hamburg, Germany) was used to capture images. Morphologic analysis of Wright-Giemsa stained cytospin preparations was assessed via a Zeiss Axioscope A1 microscope and an Axiocamera 5s with Zeiss immersol medium, and images were processed with the Zen 2.6 lite (blue) software (Zeiss, Jena, Germany).

## Supplementary Tables

### Supplementary Table S1. Characteristics of CMML patients.

| CMML  Sample | Experiment (Figure) | Diagnosis | Age at  diagnosis | Sex | 2^nd^ diagnosis | Cyto-genetics | Mutations patient | Mutations 2^nd^ Tx | Treatment | Survival  status | OS |
| --- | --- | --- | --- | --- | --- | --- | --- | --- | --- | --- | --- |
| CMML#1-MN1 | Fig.1  Fig.2 A-D  Fig.3  Fig.4  Fig.5 A-G,I  Fig.S1 A-D  Fig.S5  Fig.S7 | MDS RAEB-1 | 74 | male | CMML-1 | 46, XY | BCOR c.4144G>T; p.Glu1382Ter  DNMT3A c.2645G>A; p.Arg882His  U2AF1 c.101C>T; p.Ser34Phe  NRAS c.35G>T; p.Gly12Val  NRAS c.35G>A; p.Gly12Asp  NOTCH1 c.7169_7170insTGAGGATGGTT; p.Leu2390PhefsTer36 | BCOR c.4144G>T; p.Glu1382Ter  DNMT3A c.2645G>A; p.Arg882His  U2AF1 c.101C>T; p.Ser34Phe  NRAS c.35G>A; p.Gly12Asp  NOTCH1 c.7169_7170insTGAGGATGGTT; p.Leu2390PhefsTer36 | Azacitidine | alive | 0.54 |
| CMML#2-MN1 | Fig.2 E,F  Fig.S2 A-F | CMML-2 | 77 | male | NA | 45, X,-Y | SRSF2 c.284C>A; p.Pro95His  TET2 c.1648C>T; p.Arg550Ter  TET2 c.2428C>T; p.Gln810Ter  NRAS c.35G>C; p.Gly12Ala  DNMT3A c.2332G>A; p.Val778Met  DNMT3A c.1637_1638insA; p.Leu547AlafsTer31 | SRSF2 c.284C>A; p.Pro95His  TET2 c.1648C>T; p.Arg550Ter  TET2 c.2428C>T; p.Gln810Ter  DNMT3A c.2332G>A; p.Val778Met  DNMT3A c.1637_1638insA; p.Leu547AlafsTer31  STAG2 c.3097C>T; p.Arg1033Ter | Azacitidine | alive | 4,78 |
| CMML#3-MN1 | Fig.2 G,H  Fig.5 H  Fig.S3 A-F  Fig.S4 | CMML-1 | 72 | male | NA | 46 XY | CBL c.1259G>A; p.Arg420Gln  IDH2 c.419G>A; p.Arg140Gln  SRSF2 c.284_307del; p.Pro95_Arg102del  ASXL1 c.3015dupT; p.Glu1006Ter | CBL c.1259G>A; p.Arg420Gln  IDH2 c.419G>A; p.Arg140Gln  SRSF2 c.284_307del; p.Pro95_Arg102del  NPM1 c.860_863dupTCTG;  p.Trp288CysfsTer12 | Decitabine | alive | 0.58 |
| CMML#4 | Fig.5 H,I | CMML-1 | 69 | male | AML | 46, XY | CKIT c.2447A>T; p.Asp816Val  SRSF2 c.284C>A; p.Pro95His | Not applicable | Decitabine | dead | 2.79 |
| CMML#5 | Fig.5 H,I | CMML-1 | 66 | male | AML | 46, XY | CBL c.1151G>A; p.Cys384Tyr  SRSF2 c.284C>T; p.Pro95Leu  TET2 c.4240C>T; p.Gln1414Ter | Not applicable | CPX-351,  alloHCT | alive | 2.46 |
| CMML#6 | Fig.5 H,I | CMML-1 | 62 | male | AML | 46, XY | ASXL1 c.1934dupG; p.Gly646TrpfsTer12  SRSF2 c.284C>A; p.Pro95His | Not applicable | Azacitidine, alloHCT | alive | 4.87 |
| CMML#7 | Fig.5 H | CMML-1 | 69 | male | NA | ND | JAK2 c.1849G>T; pV617F  ASXL1 c.2957A>G; p.N986S | Not applicable | alloHCT | alive | 3.29 |

### Supplementary Table S1 continued.

| CMML  Sample | WBC at CMML diagnosis (x109/L) | Neutrophils at CMML diagnosis (absolute/µl; %) | Monocytes at CMML diagnosis (absolute/µl; %) | Blasts in PB at CMML diagnosis (%) | Hemoglobin at CMML diagnosis (g/dL) | Platelets at CMML diagnosis (x109/L) | Blasts in bone marrow at CMML diagnosis (%) |
| --- | --- | --- | --- | --- | --- | --- | --- |
| CMML#1-MN1 | 6.0 | 1.9; 31.3 | 1.7; 28.3 | 0.0 | 10.2 | 15.0 | 9.0 |
| CMML#2-MN1 | 6.5 | 38; 59.2 | 1.1; 16.5 | 13.6 | 8.4 | 20.0 | 3.0 |
| CMML#3-MN1 | 23.1 | 11.2; 48.5 | 3.9; 16.8 | 0.5 | 9.7 | 272.0 | 5.0 |
| CMML #4 | 23.6 | 11.56; 49 | 2.36; 10 | 0.0 | 14.8 | 81.0 | 7.0 |
| CMML #5 | ND | ND | ND | ND | ND | ND | 8.0 |
| CMML #6 | 44.7 | 23.7; 53 | 10.7; 24 | 1.0 | 14.4 | 136.0 | 6.0 |
| CMML#7 | 37.1 | 24.4; 65.7 | 4.1; 11.1 | 0.0 | 9.0 | 90.0 | 8.0 |

Abbreviation: NA, not applicable; ND, no data.

### Supplementary Table S2. Technical details on serial transplantations.

| Patient | 1^st^ Tx  Cell origin | Cell origin and number of retransplanted cells (x10^6^/mouse) | | | | |
| --- | --- | --- | --- | --- | --- | --- |
|  |  | 1^st^ Tx  CD3 negative CMML cells  (x10^6^/mouse) | 2^nd^ Tx | 3^rd^Tx | 4^th^Tx | 5^th^Tx |
| CMML#1 | Patient bone marrow | Untransduced (1.0)  EGFP (1.0)  MN1 (1.0) | Bone marrow from 1^st^  Tx MN1 (0.8) (fresh cells) | Bone marrow + spleen from 2^nd^ Tx MN1 (1.6)  (frozen cells) | Bone marrow + Spleen from 3^rd^ Tx MN1 (1.2) (frozen cells) | Bone marrow + Spleen from 4^th^ Tx MN1 (1.5) (frozen cells) |
| CMML#2 | Patient peripheral blood | Untransduced (1.0)  EGFP (1.0)  MN1 (1.0) | Bone marrow + spleen from 1^st^ Tx MN1 (1.2) (fresh cells) | Bone marrow + spleen from 2^nd^ Tx MN1 (1.1) (fresh cells) | NA | NA |
| CMML#3 | Patient peripheral blood | Untransduced (1.3)  MN1 (1.3) | Bone marrow + spleen from 1^st^ Tx MN1 (1.5)  (fresh cells) | Bone marrow + spleen from 2^nd^ Tx MN1 (1.0)  (fresh cells) | NA | NA |

Abbreviation: Tx, transplantation; NA, not applicable.

### Supplementary Table S3. Sequences of primers used for validation of shRNA knockdown.

| Gene | Forward primer | Reverse primer |
| --- | --- | --- |
| *ABL1* | TGGAGATAACACTCTAAGCATAACTAAAGGT | GATGTAGTTGCTTGGGACCCA |
| *BCOR* | CTGTGAGCGTGCAATGATGC | CTCGCATCTCTCACTTTCGTTC |
| *DNMT3A* | CGCATTGTGTCTTGGTGGAT | CATGGGCTGCTTGTTGTACG |
| *GATA2* | CCCACCTACCCCTCCTATGT | GCCTTCTGAACAGGAACGAG |
| *MN1* | CAAAGAAGCCCACGACCTC | CGTCACCCACGTCGTCTG |
| *NF1* | ATGGCTCTGGCCAATGTG | CAAGCTGTTGCCTCGGAAG |
| *NOTCH1* | GTGAAGGCCTCGCTGCTC | GTGGCACTCTGGAAGCACT |
| *NRAS* | TCCAGAACCACTTTGTAGATGAA | CCTTCGCCTGTCCTCATGTA |
| *U2AF1* | CGGAAAAGGCTGTGATTGAC | GTGTGCATTCTCCCATCTCA |

### Supplementary Table S4. Variant allele frequency of identified mutations from bone marrow cells from CMML#1-MN1 xenografts and bone marrow cells from the corresponding CMML#1 patient.

| Gene | Mutation | Variant allele frequency (%) mean±SEM | | | | | |
| --- | --- | --- | --- | --- | --- | --- | --- |
|  |  | Patient | 1^st^Tx | 2^nd^Tx | 3^rd^Tx | 4^th^Tx | 5^th^Tx |
| *BCOR* | c.4144G>T; p.Glu1382Ter | 45.04 | 47,70 | 49.60 | 49.98 | 49.59 | 49.82 |
| *DNMT3A* | c.2645G>A; p.Arg882His | 45.28 | 44,22 | 48.73 | 49.36 | 47.33 | 48.74 |
| *NOTCH1* | c.7169_7170insTGAGGATGGTT; p.Leu2390PhefsTer36 | 14 | 46.32 | 50.10 | 50.18 | 48.49 | 52.38 |
| *NRAS* | c.35G>T; p.Gly12Val | 21.3 | 0,41 | 0 | 0 | 0 | 0 |
| *NRAS* | c.35G>A; p.Gly12Asp | 17.77 | 48.93 | 46.97 | 49.29 | 51.82 | 50.61 |
| *U2AF1* | c.101C>T; p.Ser34Phe | 44.56 | 51.82 | 54.29 | 42.81 | 45.15 | 46.47 |

Abbreviation: Tx, transplantation.

### Supplementary Table S5. Sequences of shRNAs directed against recurrently mutated genes.

| shRNA | Sequence |
| --- | --- |
| shMN1-2049 | ACGGGCTTCTAGTCCGACAAAATAGTGAAGCCACAGATGTATTTTGTCGGACTAGAAGCCCGG |
| shMN1-2165 | AACGCGCAATTCGAGTATCCCATAGTGAAGCCACAGATGTATGGGATACTCGAATTGCGCGTG |
| shNRAS-620 | CAGGACAGTTGATACAAAACAATAGTGAAGCCACAGATGTATTGTTTTGTATCAACTGTCCTT |
| shNRAS-1670 | ACAGGAGAAAGATGAAACTGAATAGTGAAGCCACAGATGTATTCAGTTTCATCTTTCTCCTGG |
| shU2AF1-116 | ACACCGAGAAAGACAAAGTCAATAGTGAAGCCACAGATGTATTGACTTTGTCTTTCTCGGTGC |
| shU2AF1-595 | CCGCAAGAAGCATAGATCAAGATAGTGAAGCCACAGATGTATCTTGATCTATGCTTCTTGCGA |
| shNOTCH1-8397 | CCAGGTTCAGTATTATGTAGTTTAGTGAAGCCACAGATGTAAACTACATAATACTGAACCTGA |
| shNOTCH1-9178 | CTAGAGTGTAGTTTACAGAAAATAGTGAAGCCACAGATGTATTTTCTGTAAACTACACTCTAT |
| shDNMT3A-1548 | ACCGGCTCTTCTTTGAGTTCTATAGTGAAGCCACAGATGTATAGAACTCAAAGAAGAGCCGGC |
| shDNMT3A-3221 | CCCCTTTGATTGTTTTCTAAAATAGTGAAGCCACAGATGTATTTTAGAAAACAATCAAAGGGT |
| shBCOR-4253 | AACGGAGACTTATTGTCAATAATAGTGAAGCCACAGATGTATTATTGACAATAAGTCTCCGTG |
| shBCOR-5175 | ACACCATGTACAGTGTGTTATATAGTGAAGCCACAGATGTATATAACACACTGTACATGGTGG |
| shGATA2-176 | CCGAGGTGGACGTCTTCTTCAATAGTGAAGCCACAGATGTATTGAAGAAGACGTCCACCTCGT |
| shGATA2-3007 | ATGAGTACTGTTAAGAATAATATAGTGAAGCCACAGATGTATATTATTCTTAACAGTACTCAC |
| shNF1-896 | AAATGTTGATGTTCATGATATATAGTGAAGCCACAGATGTATATATCATGAACATCAACATTG |
| shNF1-1578 | ATGGCTCAGAATTCACCTTCTATAGTGAAGCCACAGATGTATAGAAGGTGAATTCTGAGCCAG |
| shRen-713 (CTL) | CAGGAATTATAATGCTTATCTATAGTGAAGCCACAGATGTATAGATAAGCATTATAATTCCTA |

### Supplementary Table S6. Genes covered by our custom myeloid panel (based on GRCh37/hg19).

| Gene | Exons | Gene | Exons | Gene | Exons |
| --- | --- | --- | --- | --- | --- |
| ASXL1 | 12 | GATA2 | 2-6 | RUNX1 | complete |
| ASXL2 | 11+12 | IDH1 | 4 | SETBP1 | 4 |
| BCOR | complete | IDH2 | 4 | SF3B1 | 13-16 |
| BCORL1 | complete | JAK2 | 12, 14 | SMC1A | 2, 11, 16, 17 |
| BRAF | Exon15 | KDM6A | complete | SMC3 | 10, 13, 19, 23, 25, 28 |
| CALR | 9 | KIT | 2, 8-11, 13, 17 | SRSF2 | 1 |
| CBL | 8, 9 | KRAS | 2-5 | STAG1 | complete |
| CEBPA | complete | MPL | 10 | STAG2 | complete |
| CSF3R | 14-17 | MYC | 2 | TET2 | 3-11 |
| CSNK1A1 | 3, 4 | NF1 | complete | TP53 | 2-11 |
| DDX41 | complete | NPM1 | 11 | U2AF1 | 2, 6 |
| DNMT3A | complete | NRAS | 2-5 | WT1 | 7, 9 |
| ETNK1 | 3 | PHF6 | complete | ZBTB7A | 2, 3 |
| ETV6 | complete | PPM1D | 1-6 | ZRSR2 | complete |
| EZH2 | complete | PTPN11 | 3, 13 |  |  |
| FLT3 | 14-16, 20 | RAD21 | complete |  |  |

### Supplementary Table S7. Primers used for error corrected sequencing.

| Primer name | Forward | Reverse |
| --- | --- | --- |
| NRAS_NGS_1 | GCTCGCCAATTAACCCTGAT | AGTGGTTCTGGATTAGCTGGA |
| U2AF1_NGS_31 | CCCAGCAAAATAATCAGCTCTCA | ACAAACCTGGCTAAACGTCG |
| DNMT3A_NGS_882 | GGTATTTGGTTTCCCAGTCC | GAAGAGGTGGCGGATGACT |
| BCOR_NGS_1375_1391 | GAGAAGCCATCCGGGAAGAG | GGGTCAAGAGGTACCTTGCC |
| NOTCH1_NGS_2383_2412 | GATGATGAGCTACCAGGGCC | CTGCTGCTGGATGTTTGCTG |

### Supplementary Table S8. Sequences of primers used for amplicon-based next generation sequencing.

| PCR | Primer sequence |
| --- | --- |
| 1. Lentivirus  backbone | EGFP forward: CAAGATCCGCCACAACATCG  WPRE reverse: CCACATAGCGTAAAAGGAGCAAC |
| 2. miR30N+  common  sequence | CS3-miR30N F: GGTAAACACAAGGGCACTGGGATTACTTCTTCAGGTTAACCCAACAG  CS4-miR30N R: CGGACTACAGCTCCCATCATTGCTCCTAAAGTAGCCCCTTGAAGTCC |
| 3. Multiplex  Identifier (MID,barcode) | e.g. MID1-CS3 F: AATGATACGGCGACCACCGAGATCTACACTCTTTCCCTACACGACG  CTCTTCCGATCTAACGAGTGCGTGGTAAACACAAGGGCACTGG  e.g.MID2-CS4 R: CAAGCAGAAGACGGCATACGAGATACATCTAGTGGCTCAGAGTTCTAC  AGTCCGACG ATCATACGCTCGACACGGACTACAGCTCCCATCAT |

## Supplementary Figures

### Supplementary Figure S1. MN1 expression is required for engraftment of human CMML cells.

1. Transduction efficiency of EGFP-transduced (CMML#1-EGFP) and MN1-transduced (CMML#1-MN1) primary CMML cells prior transplantation in NSGS recipient mice.
2. Representative flow cytometric analysis of cells isolated from blood, bone marrow and spleen of NSGS recipient mice 13 weeks after transplantation indicating CMML#1-MN1 engraftment by hCD45+EGFP+ cells.
3. Light and fluorescence microscopy from bone marrow smears of a CMML#1-MN1 mouse, in which CMML#1-MN1 cells engrafted as indicated by EGFP fluorescence.
4. Immunophenotype of engrafted CMML#1-MN1 cells in bone marrow and spleen of a representative secondary recipient mouse.


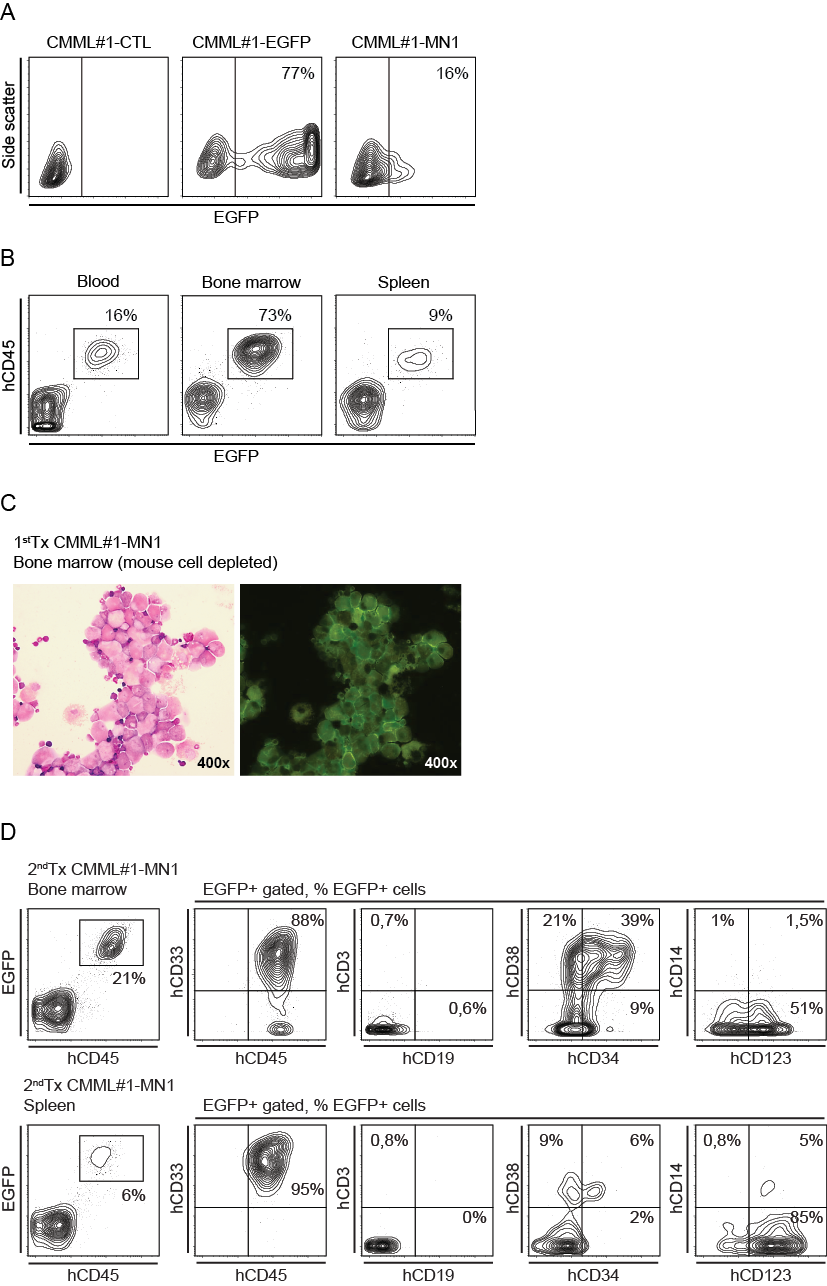


### Supplementary Figure S2. Robust engraftment of CMML#2-MN1 cells in vivo.

1. Engraftment of CMML#2 cells (human CD45+) in the peripheral blood, bone marrow and spleen of secondary recipient NSGS mice (number of analyzed mice is indicated in the figure; mean ± SEM).
2. Spleen weight of CMML#2 secondary recipient NSGS mice at sacrifice (number of analyzed mice is indicated in the figure; mean ± SEM).
3. Platelet count in peripheral blood of CMML#2 secondary recipient NSGS mice at sacrifice (number of analyzed mice is indicated in the figure; mean ± SEM).
4. White blood cell count in peripheral blood of CMML#2 secondary recipient NSGS mice at sacrifice (number of analyzed mice is indicated in the figure; mean ± SEM).
5. Hemoglobin level in peripheral blood of CMML#2 secondary recipient NSGS mice at sacrifice (number of analyzed mice is indicated in the figure; mean ± SEM).
6. Morphology from bone marrow smears of the CMML#2 patient at diagnosis and from a CMML#2 secondary recipient mouse at sacrifice.


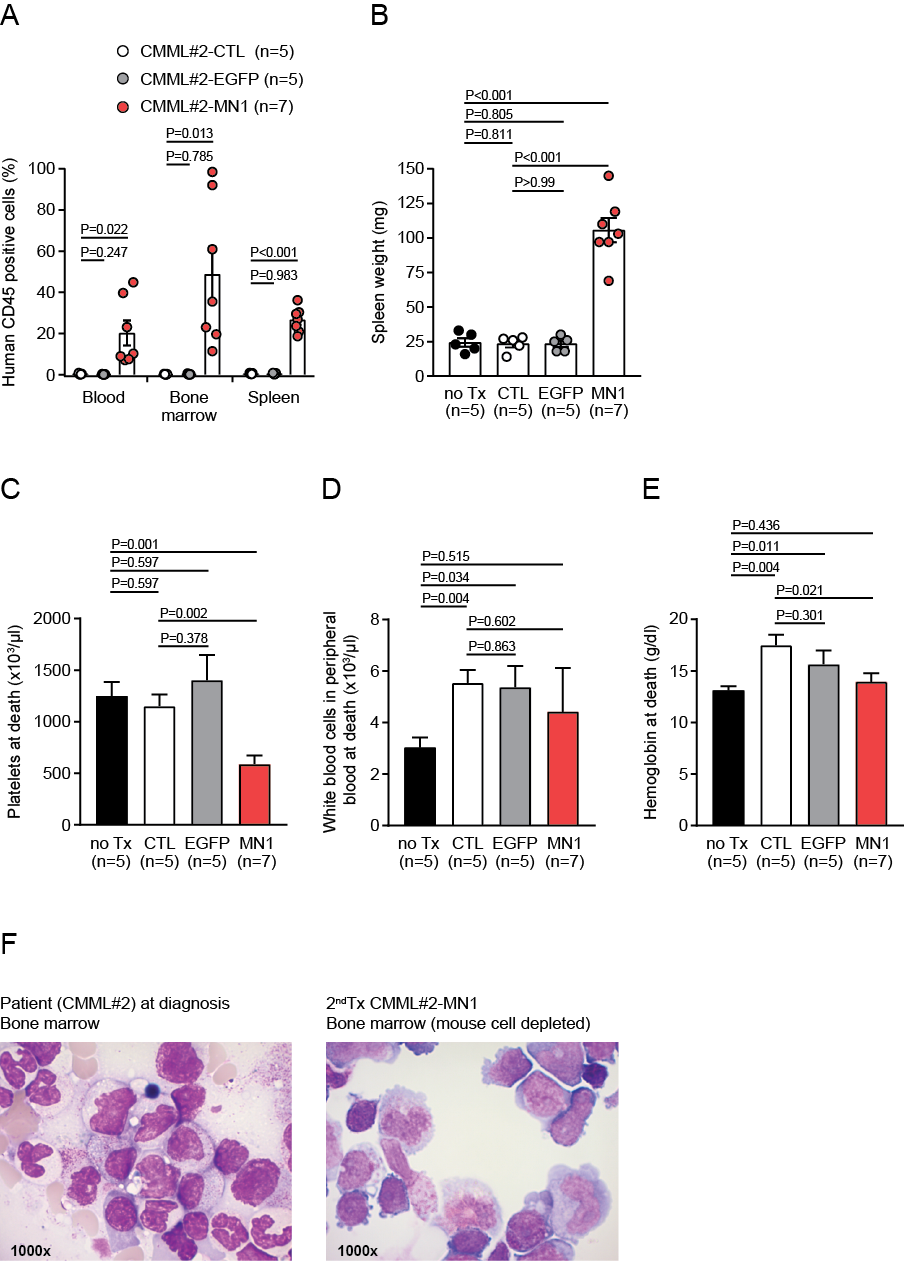


### Supplementary Figure S3. Robust engraftment of CMML#3-MN1 cells in vivo.

1. Engraftment of CMML#3 cells (human CD45+) in the peripheral blood, bone marrow and spleen of secondary recipient NSGS mice (number of analyzed mice is indicated in the figure; mean ± SEM).
2. Spleen weight of CMML#3 secondary recipient NSGS mice at sacrifice (number of analyzed mice is indicated in the figure; mean ± SEM)
3. Platelet count in peripheral blood of CMML#3 secondary recipient NSGS mice at sacrifice (number of analyzed mice is indicated in the figure; mean ± SEM).
4. White blood cell count in peripheral blood of CMML#3 secondary recipient NSGS mice at sacrifice (number of analyzed mice is indicated in the figure; mean ± SEM).
5. Hemoglobin level in peripheral blood of CMML#3 secondary recipient NSGS mice at sacrifice (number of analyzed mice is indicated in the figure; mean ± SEM).
6. Morphology from bone marrow smears of the CMML#3 patient at diagnosis and from a CMML#3 secondary recipient mouse at sacrifice.


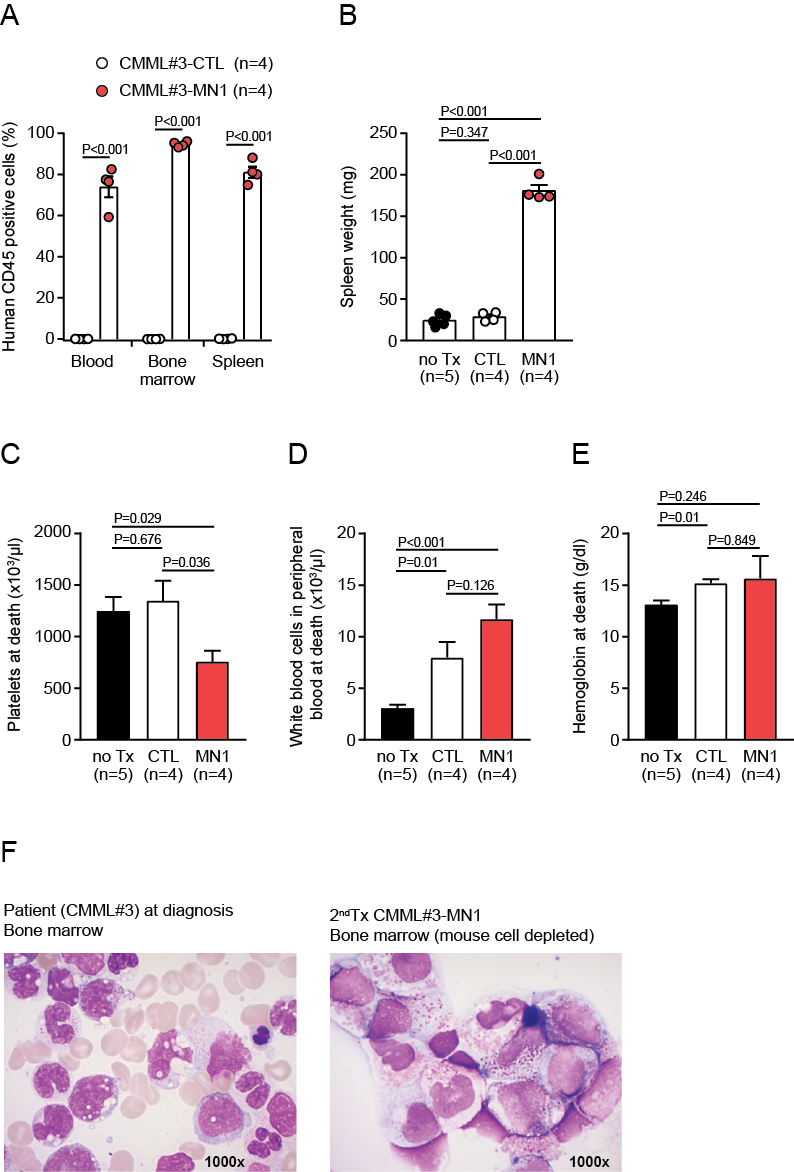


### Supplementary Figure S4. Clonal selection of CMML#3-MN1 cells in vivo.

Variant allele frequencies of mutated genes in cells from the CMML#3 patient, corresponding CMML#3-MN1 cells before transplantation and CMML#3-MN1 cells engrafted in bone marrow of NSGS recipient mice.


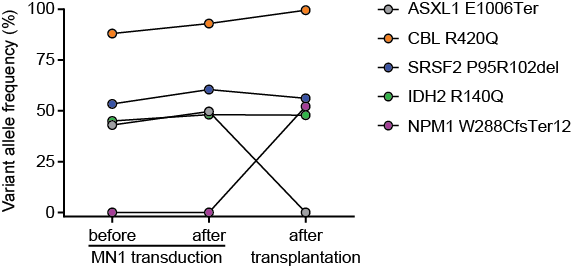


### Supplementary Figure S5. Engraftment of CMML#1-MN1 cells is enhanced by GM-CSF.

Engraftment kinetics of CMML#1-MN1 cells from 4^th^ transplantation in NSG or NSGS recipient mice at the indicated time points (* indicates time of sacrifice; n=4, mean ± SEM).


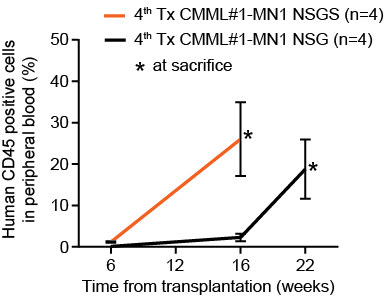


### Supplementary Figure S6. Validation of shRNAs directed against recurrently mutated genes.

Relative gene expression quantified by qRT-PCR in K562 cells transduced with shRNAs targeting the genes *MN1, NRAS, U2AF1, NOTCH1, DNMT3A, BCOR, GATA2* and *NF1*. Expression levels are relative to the *ABL1* housekeeping gene and normalized to gene expression in K562 cells transduced with a shRNA targeting the luciferase gene (shCTL) (n=3, mean± SEM).


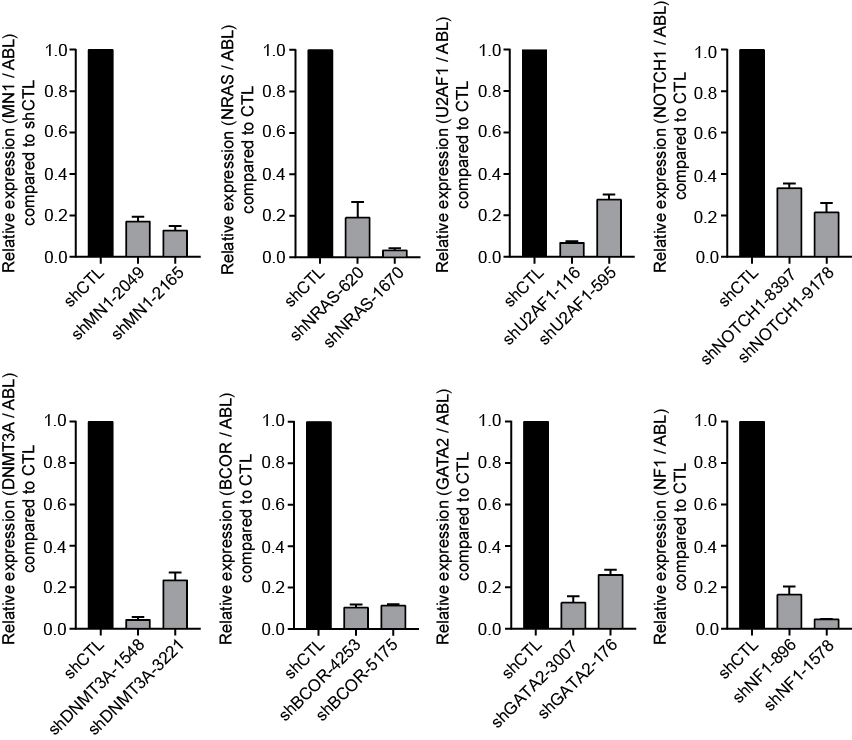


### Supplementary Figure S7. The MEK-inhibitor trametinib reduces engraftment of CMML#1-MN1 cells in vivo when combined with azacitidine.

1. Representative flow cytometric analysis of cells from peripheral blood of NSGS recipient mice 2 weeks after transplantation indicating CMML#1-MN1 engraftment by human CD45+EGFP+ cells.
2. Engraftment of CMML#1-MN1 cells in peripheral blood of mice treated with vehicle, trametinib, azacitidine and the combination of trametinib+azacitidine at the indicated time points (arrow indicates start of treatment, number of analyzed mice is indicated in the figure, mean ± SEM).
3. Engraftment of CMML#-MN1 cells in blood, bone marrow and spleen of recipient mice treated with vehicle, trametinib, azacitidine and the combination of trametinib+azacitidine at sacrifice (number of analyzed mice is indicated in the figure, mean ± SEM).


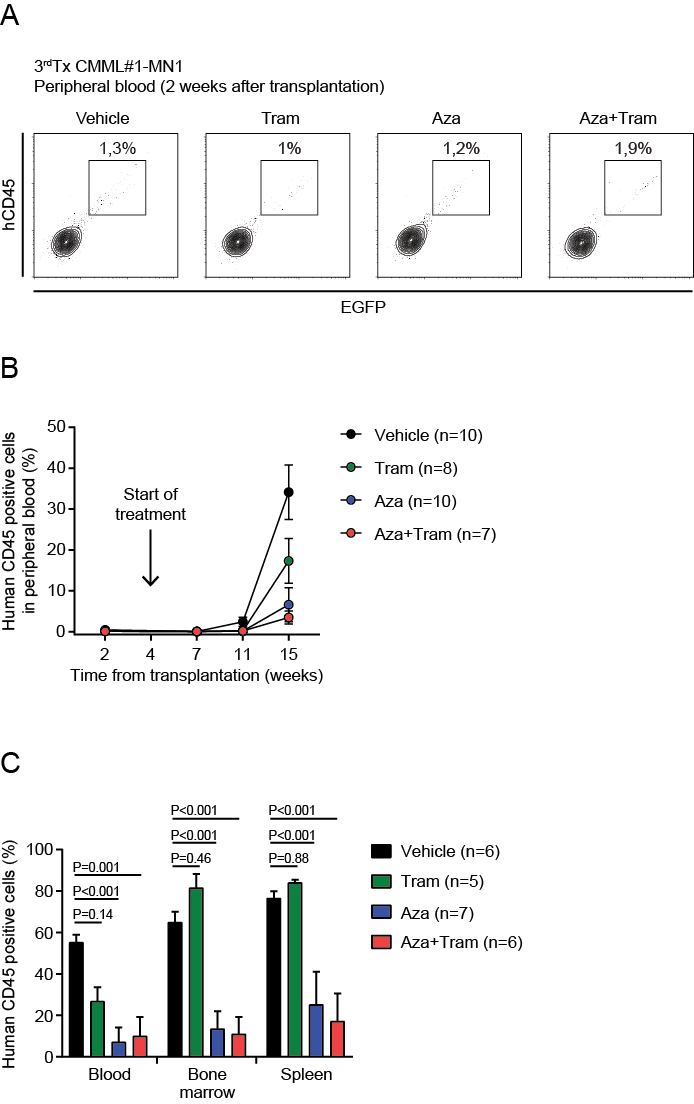


## Supplementary References

1. Adams FF, Heckl D, Hoffmann T, Talbot SR, Kloos A, Thol F, et al. An optimized lentiviral vector system for conditional RNAi and efficient cloning of microRNA embedded short hairpin RNA libraries. Biomaterials. 2017;139:102-15.

2. Heuser M, Gabdoulline R, Loffeld P, Dobbernack V, Kreimeyer H, Pankratz M, et al. Individual outcome prediction for myelodysplastic syndrome (MDS) and secondary acute myeloid leukemia from MDS after allogeneic hematopoietic cell transplantation. Annals of hematology. 2017;96(8):1361-72.

3. Thol F, Gabdoulline R, Liebich A, Klement P, Schiller J, Kandziora C, et al. Measurable residual disease monitoring by NGS before allogeneic hematopoietic cell transplantation in AML. Blood. 2018;132(16):1703-13.

4. Miller CA, McMichael J, Dang HX, Maher CA, Ding L, Ley TJ, et al. Visualizing tumor evolution with the fishplot package for R. BMC genomics. 2016;17(1):880.
